# Medical Qualification and Hospital Appointments

**Published:** 1907-06-29

**Authors:** 


### The Hospital

A Journal of

The Medical Sciences and Hospital Administration.

New Series. No. 14, Vol. I. [No. 1086, Vol. XLII.] SATURDAY, JUNE 29, 1907.

### MEDICAL QUALIFICATION AND HOSPITAL APPOINTMENTS.

It cannot be questioned that one of the duties incumbent on the governing authorities of any hospital is to obtain for the patients of their institution the benefit of the highest available medical and surgical skill. To neglect this claim is to be false to a great and responsible trust. Thus it becomes an important matter to determine by what standard applicants for positions on the staffs of hospitals shall be tested. Whatever standard be adopted, it may be taken for granted that in occasional instances it will prove more or less unsatisfactory, and that, human judgment being fallible, mistakes will at times be made. Yet, for practical working purposes, and because the number of applicants will probably always be in excess of the number of appointments, some method of appraisement must be adopted. In this country, for the most part, hospital authorities demand of any candidate for a position on the hospital staff the possession of one of the higher diplomas granted by a College of Physicians or College of Surgeons. This condition has several advantages. In the first place, it ensures that the candidate has attained a certain level of professional knowledge, so far as this can be tested by examination; secondly, and more important, it guarantees a period of special study devoted, as the case may be, either to medicine on the one hand, or to surgery on the other; and yet again, it brings every member of the hospital staff under certain special collegiate rules which tend to the cultivation of a high standard of professional conduct. Hence the enforcement of the condition here alluded to enables the governing committee of a hospital to satisfy themselves that they are adopting a reasonable method to maintain the reputation and efficiency of the hospital. No one with any practical knowledge of affairs will argue that the mere passing of an examination is a proof of intellectual superiority, and still less that it is a guarantee of good judgment and common sense. But in the case now in question it has the results just described, and hence at least an *a priori* case may be made out in its favour.

It happens that within recent weeks an attack on the custom prevailing in regard to the appointment of hospital staffs has appeared in the form of a letter signed by the President of the Association of Scottish Medical Diplomates. In this it is argued that the restrictive conditions prevalent in England, and particularly in London, are unjust to the diplomates of the Scottish colleges, and are prejudicial to the public interest as they narrow the field of competition and "close the door to many a fine career." From the terms of the letter it appears that the claim is made for all "Scottish diplomates," and is not restricted to the holders of the so-called higher qualifications. We certainly have no word to say against the qualifications granted by the Scottish colleges, but it is obvious that the demand advanced in the letter is only partial and incomplete. If it to be granted, an equal opportunity must necessarily be given to all those who hold licenses to practise from the English colleges. For it would surely be the height of absurdity that a Scottish diploma should open the way to the staff of an English hospital, while an English diploma of equal rank should have no virtue for such a purpose. Therefore, if the Scottish Association is to maintain its position it must widen the basis of its claim and must demand that hospital appointments in England be open to the candidature of all registered medical practitioners. Hence the first thing for it to do is to show that the English diplomates generally support such a demand, and before taking the field here it would be well for the Association to assure itself that the restrictions which it denounces have no counterpart in the hospitals north of the Tweed. Until it can satisfy these two conditions the demand of the Association as now presented will hardly produce much effect.

Assuming, however, that what the Association really desires, is to maintain the existing restrictions as against a mere qualification to practise, but to relax them in regard to the higher qualifications of the Scottish colleges, it may be asked whether they present any better case than the one just discussed. We venture to reply in the negative. Such a position involves the admission that something more than a qualifying diploma ought to be demanded from those who aspire to the responsibilities of a hospital appointment. And if this is the case, it is surely not unnatural that in England the special guarantee should be that afforded by the English colleges. We question very much whether Scottish graduates have any complaint to make of monopoly and injustice. To speak of "disqualification of all Scottish competitors," as is done in the Association's letter, is to suggest a state of matters which does not exist. The positions occupied by Scottish graduates in the medical world of London affords a prompt contradiction to such a statement. In short the Association has no authority to speak for the general body of the profession in England, and to claim a special preference for Scottish diplomates is an absurdity. We question, further, whether any considerable number of Scotsmen have any sympathy with such a proposal.

